# Wastewater-Based Surveillance Does Not Belong in a Regulatory Framework Designed to Protect Waters That Receive Treated Wastewater. Comment on Wright, T.; Adhikari, A. Utilizing a National Wastewater Monitoring Program to Address the U.S. Opioid Epidemic: A Focus on Metro Atlanta, Georgia. *Int. J. Environ. Res. Public Health* 2023, *20*, 5282

**DOI:** 10.3390/ijerph20176636

**Published:** 2023-08-24

**Authors:** Anna Mehrotra, Naoko Munakata, Rasha Maal-Bared, Daniel Gerrity, Jennifer Sabater, Scott Bessler

**Affiliations:** 1 Water Environment Federation, Alexandra, VA 22314, USA; jsabater@wef.org; 2 Los Angeles County Sanitation Districts, Whittier, CA 90601, USA; nmunakata@lacsd.org; 3 EPCOR Water Services Inc., Edmonton, AB T5J 3Y3, Canada; rmaal-bared@epcor.com; 4 Southern Nevada Water Authority, Las Vegas, NV 89193, USA; daniel.gerrity@snwa.com; 5 Metropolitan Sewer District of Greater Cincinnati, Cincinnati, OH 45204, USA; scott.bessler@cincinnati-oh.gov

We read with great interest the work by Wright and Adhikari on “Utilizing a National Wastewater Monitoring Program to Address the U.S. Opioid Epidemic: A Focus on Metro Atlanta, Georgia” [1]. We appreciate the focus on the opioid health crisis and the discussion of wastewater testing as a public health tool. Still, we would like to take the opportunity to caution article readers about the authors’ primary recommendation to mandate wastewater-based surveillance (WBS) of opioids via National Pollutant Discharge Elimination System (NPDES) permits. WBS, which focuses on testing *untreated influent* wastewater as a means of informing public health action, does not belong in a regulatory framework designed to protect receiving waters based on *treated effluent* quality. The existing evidence indicates that receiving waters are not at risk from opioid compounds and their metabolites that might be present in treated effluent. Further, requiring analysis of opioid compounds at the water resource recovery facility (WRRF), rather than upstream/sub-sewershed locations, fails to achieve the granularity needed to maximize public health benefits in the fight against opioid abuse. Instead, this would negatively impact WRRFs and jeopardize their critical support of WBS programs—support that has already been successfully demonstrated through national WBS programs that rely on voluntary participation of wastewater agencies in Australia [2,3], Canada [4,5], the European Union [6,7], and USA [8].

WBS, also known as wastewater-based epidemiology (WBE) or wastewater monitoring for public health, can provide crucial information on the presence of viral, fungal, bacterial, and protozoan pathogens in a community [9]. Moreover, numerous studies have relied on untreated wastewater to estimate opioid and other illicit drug consumption [10,11,12]. WBS relies on collecting and testing untreated influent wastewater samples [13] because wastewater treatment processes can degrade biological and chemical markers relevant to assessing population health [14,15,16]. Accordingly, treated effluent monitoring has limited (if any) value for assessing and responding to public health conditions in the upstream community.

The NPDES permit program is intended to ensure adequate wastewater treatment before discharge into receiving water bodies. NPDES permits issued to WRRFs include limits on the levels of pollutants that can be discharged in treated effluent, as well as the monitoring and reporting required to demonstrate compliance with the treated effluent pollutant limits. Although some permits may include monitoring and reporting requirements for influent wastewater, such requirements generally pertain to the pollutants of interest in treated effluent. WBS data are not useful for monitoring permit compliance, evaluating wastewater treatment processes, or ensuring receiving water protection. Thus, this regulatory framework is inappropriate for WBS. Monitoring effluent discharges would likely provide minimal benefit or actionable information for public health professionals due to the degradation of relevant markers during treatment. In addition, WBS programs rely on input from both public health departments and WRRFs to produce the most valuable data for local public health interventions at the most suitable locations. Since the early days of the COVID-19 pandemic, many WRRFs have willingly volunteered to collaborate with health departments to create successful WBS programs by contributing samples and other resources. These programs must be flexible and adapt as conditions change, making collaboration more appropriate than a rigid, permit-driven approach.

NPDES permit limits are developed to meet water quality standards to protect human health and aquatic life in water bodies that receive pollutants discharged from point sources, including WRRFs. Adding water quality standards for new compounds such as opioids would need to follow the deliberate process that has been designed to incorporate the most credible scientific evidence on risks and ensure that new water quality objectives are implemented where appropriate [17]. While additional research is warranted, the available evidence does not support the conclusion that human health or aquatic life is at risk from opioid compounds and their metabolites in waters impacted by treated wastewater effluent. As shown in Table 1, reported concentrations of opioids and their metabolites in treated effluent are usually less than 1000 ng/L. Concentrations in receiving waters are likely to be lower (due to dilution), depending on concentrations in the water body upstream of the discharge. By comparison, one study with zebrafish (*Danio rerio*) embryos and larvae found the lowest significant behavior effect concentrations for fentanyl and tramadol to be 530,000 ng/L and 300,000 ng/L, respectively [18]. Another study, described in Taylor [19], illustrated that zebrafish will seek out hydrocodone and undergo withdrawal symptoms when the drug is removed [20]. However, the hydrocodone concentration used by the researchers was 6,000,000 ng/L, or more than 10,000 times higher than the highest concentrations reported by Campos-Manas et al. [21] in wastewater effluent, and therefore unlikely to be environmentally relevant. Establishing whether human health or aquatic life is at risk from opioids in receiving water bodies should be based on research incorporating opioid compounds and their metabolites at environmentally relevant concentrations.

We agree that WBS is ideally used in settings that enable “swift, directed interventions” for as “much of the entire population-of-interest as possible” [1]. For opioids, such settings would necessarily include upstream sampling locations at manholes and pumping or lift stations within the centralized sewer network to identify specific areas most impacted by opioid abuse. However, NPDES permit-based monitoring and reporting requirements typically apply either to the WRRF influent (which provides data at larger regional scales than upstream sampling) or to treated effluent (which is not useful for WBS due to degradation of markers throughout the treatment processes). Therefore, permit-driven monitoring would either not provide the data granularity needed for public health interventions or would generate confounded data due to the effects of wastewater treatment on opioids. In addition, while most likely to result in practical public health actions, upstream sampling is resource-intensive and raises ethical concerns that have not previously been considered in the NPDES framework [22].

**Table 1:** Range of opioid compound concentrations reported globally in wastewater treatment plant effluents.

Opioid Compound	Concentration Range Reported in Treated Effluent ^1^ (ng/L)	Reference(s)
6-Acetylmorphine (heroin metabolite)	Not detected ^2^	[23]
Codeine	3.1–~2250	[21,23,24]
EDDP (methadone metabolite)	2.7–1150	[21,23]
Fentanyl	Not detected ^3^	[21,23]
Heroin	Not detected ^4^	[23]
Hydrocodone	50–210	[21]
Methadone	3.4–730	[23]
Morphine	12–250	[23,24]
O-desmethyltramadol (tramadol metabolite)	44–760	[21]
Oxycodone	54–260	[21]
Norcodeine	5.2–40	[23]
Normorphine	31–110	[23]
Tramadol	75–1300	[21]

^1^ For samples above the reported limit of quantification or method detection limit. ^2^ Not detected in any samples from [23] (n = 15; limit of quantification = 3.1 ng/L). ^3^ Not detected in any samples from [21] (n = 25; method detection limit = 3.4 ng/L) or [23] (n = 15; limit of quantification = 1.7 ng/L). ^4^ Not detected in any samples from [23] (n = 15; limit of quantification = 20 ng/L).

Wright and Adhikari [1] call for analysis of opioids in 24-h composite wastewater samples by laboratory analysts using liquid chromatography–mass spectrometry (LC–MS) or LC–MS/MS. Doing so would require significant and potentially prohibitive new investments in either (1) laboratory equipment, since very few wastewater laboratories are currently equipped with the necessary analytical instrumentation; or (2) ongoing sample analyses by a contract laboratory. Many small WRRFs have limited resources and are often located in rural areas, where opioid abuse has been especially pronounced [25]. Therefore, updating all WRRFs to be equipped for on-site opioid analysis is impractical. Instead, most WRRFs would need to utilize a contract lab and potentially spend more than USD 1000 per sample to analyze a suite of opioids and their metabolites, equating to more than USD 100,000 per year per sampling location (assuming twice-weekly testing). Multiple sampling locations would likely be necessary in order to provide the resolution needed for effective public health interventions, potentially increasing costs exponentially. Further, for some remote WRRFs, substantial additional costs could be accrued during sample transport to a contract lab. Many WRRFs do not have the resources to afford the analysis of wastewater samples for opioids without significant cost increases to their ratepayers, particularly in the face of rising costs for labor, supplies, and potentially new compliance requirements for compounds such as PFAS. In other words, permit-driven monitoring of opioids would result in a “one-size-fits-all” approach that would be highly inefficient, and even detrimental, if implemented across all WRRFs. Instead, this tool should be implemented more strategically to balance public health benefits with incurred costs, particularly taking into consideration resource limitations in some areas.

The opioid epidemic is undoubtedly claiming too many lives, including in the Metro Atlanta area, which was the focus of Wright and Adhikari [1]. While we agree that WBS can potentially “provide real-time data to assist public health officials on how to address the opioid epidemic within their communities” [1], NPDES permit-based monitoring of WRRF influents and effluents will not generate the granular information needed to identify areas most impacted by opioid abuse. Any WBS program needs to be led by the appropriate public health agency in collaboration with utilities and other stakeholders, not mandated through a rigid permitting process that will make it challenging to develop and maintain a flexible program tailored to local conditions. The NPDES program is intended to protect receiving waters by controlling wastewater discharges. WBS is inappropriate within this framework, as WBS data are not helpful for monitoring permit compliance, evaluating treatment processes, or ensuring receiving water protection. Based on the current evidence, including opioid limits in NPDES permits is not warranted for meeting water quality standards to protect human health and aquatic life. Moreover, the additional financial burden on WRRFs to meet opioid-related permit limits would distract from their mission to treat wastewater to meet current discharge requirements. This would potentially undermine support for WBS programs, without generating highly actionable data. While WBS should play a role in addressing the opioid epidemic, WBS data can be successfully generated through a voluntary, collaborative model rather than a regulatory approach. This has already been successfully demonstrated in WBS programs throughout the world, including the National Wastewater Surveillance System (NWSS) implemented by the U.S. Centers for Disease Control and Prevention. With sufficient financial support, these existing national programs could be expanded to other targets of interest, including opioids, while ensuring sufficient flexibility to respond to rapidly changing conditions and emerging public health threats.
